# Neuroinflammation as Fuel for Axonal Regeneration in the Injured Vertebrate Central Nervous System

**DOI:** 10.1155/2017/9478542

**Published:** 2017-01-19

**Authors:** Ilse Bollaerts, Jessie Van houcke, Lien Andries, Lies De Groef, Lieve Moons

**Affiliations:** Laboratory of Neural Circuit Development and Regeneration, Animal Physiology and Neurobiology Section, Department of Biology, KU Leuven, Leuven, Belgium

## Abstract

Damage to the central nervous system (CNS) is one of the leading causes of morbidity and mortality in elderly, as repair after lesions or neurodegenerative disease usually fails because of the limited capacity of CNS regeneration. The causes underlying this limited regenerative potential are multifactorial, but one critical aspect is neuroinflammation. Although classically considered as harmful, it is now becoming increasingly clear that inflammation can also promote regeneration, if the appropriate context is provided. Here, we review the current knowledge on how acute inflammation is intertwined with axonal regeneration, an important component of CNS repair. After optic nerve or spinal cord injury, inflammatory stimulation and/or modification greatly improve the regenerative outcome in rodents. Moreover, the hypothesis of a beneficial role of inflammation is further supported by evidence from adult zebrafish, which possess the remarkable capability to repair CNS lesions and even restore functionality. Lastly, we shed light on the impact of aging processes on the regenerative capacity in the CNS of mammals and zebrafish. As aging not only affects the CNS, but also the immune system, the regeneration potential is expected to further decline in aged individuals, an element that should definitely be considered in the search for novel therapeutic strategies.

## 1. Introduction

Brain injuries and neurodegenerative disorders, such as Alzheimer's and Parkinson's disease, multiple sclerosis, or glaucoma, represent a growing social and economic burden and affect an increasing number of people in our aging society. Traumatic lesions and neurodegeneration drastically reduce life quality and lead to severe and often fatal impairments, largely because the central nervous system (CNS) of adult mammals retains only little capacity for regeneration into adulthood, which comprises the replacement of lost neurons (de novo neurogenesis) and/or the repair of damaged axons (axonal regeneration) [1, 2]. The lack of long-distance axonal regeneration in the mature mammalian CNS, on which will be focused here, has been attributed to an insufficient intrinsic growth capacity of its neurons and an inhibitory extrinsic environment [3, 4]. Our current knowledge of the underlying molecules and pathways mainly comes from two well characterized rodent injury models: optic nerve and spinal cord lesions. Damage to the optic nerve, which solely consists of axons originating from the retinal ganglion cells (RGC) located in the inner retina, results in apoptotic RGC death and consequently in vision loss [5–7]. Preservation of injured cells followed by axonal regeneration can be stimulated, both by intrinsic and by extrinsic factors, but full functional recovery has not yet been achieved [8–10]. Spinal cord injuries lead to death of the damaged cells at the epicenter of the lesion, including neurons, oligodendrocytes, and astrocytes. After the primary insult, secondary processes (excitotoxicity, oxidative stress, etc.) may cause additional loss of neurons and supporting cells. Furthermore, interrupted descending and ascending axonal tracts have debilitating consequences, and although proximal segments typically survive, they do not regenerate spontaneously [11–13]. Restoration of motor and sensory tracts via axonal regeneration is believed to be the most promising way to reverse paralysis after spinal cord injury [14]. Regenerative strategies known thus far, as well as identified intracellular pathways and growth-inhibiting factors, are largely similar to those characterised in optic nerve regeneration [15, 16].

In mammals, the acute inflammatory response that takes place rapidly after traumatic CNS lesions is put forward as one of the major elements affecting the regenerative outcome [17]. Microglia, the main mediators of inflammation in the CNS, are among the first cells to respond to damage. They become activated, thereby changing their morphology from ramified to amoeboid, proliferate, migrate to the injury site, and start to produce a variety of pro- and anti-inflammatory cytokines [18]. Furthermore, neutrophils and macrophages from the periphery are recruited to the injured area, and, together with reactive astrocytes, microglia/macrophages will contribute to the formation of a regeneration-inhibiting glial scar [4, 19]. Traditionally, the acute inflammatory response has been viewed as a detrimental orchestrator in CNS damage and pathology. After spinal cord injury, depletion of peripheral macrophages enhances axonal regeneration and improves functional recovery [20]. Administration of the anti-inflammatory drug minocycline gives similar results [21]. However, more recent evidence suggests that the inflammatory response can also positively contribute to regeneration [22, 23], as is exemplified by an improved behavioural outcome after spinal cord injury resulting from an increased number of monocyte-derived macrophages via adoptive transfer [24]. These conflicting results have led to substantial controversy regarding the negative or positive effect of acute inflammation in CNS regeneration. Ongoing and future investigations of its mediator cells and their key regulatory switches thus seem to be essential for a better understanding of how successful regeneration can be induced.

In sharp contrast to mammals, adult zebrafish are capable of extensive and successful regeneration throughout their body, including their fins, heart, liver, and CNS [25, 26]. This has sparked the interest of many neuroscientists, who turned to this small laboratory animal to understand the crucial molecules underlying successful CNS repair. Adult zebrafish retain the capacity of robust axonal regeneration and can morphologically and functionally recover from optic nerve and spinal cord injuries [27]. Moreover, similar to the situation in mammals, an acute inflammatory response occurs after CNS injury in zebrafish, which has recently been suggested to positively contribute to the regenerative process [28, 29]. Given the high degree of conservation between teleosts and mammals at both the molecular and genetic level, research in zebrafish can help to overcome the limitations of nonregenerative mammalian models, in which the regenerative outcome is always suboptimal, even when stimulated [25]. A thorough understanding of how zebrafish can couple acute inflammation to successful regeneration after injury may thus contribute to the development of regenerative therapies.

Since neurodegenerative diseases are age-related, regenerative therapies will often need to be achieved in the senescent CNS. Therefore, the effect of aging on the regenerative potential should not be overlooked. Indeed, aging processes affect CNS functioning, as is evidenced by, for instance, a reduced synaptic efficacy and neuronal loss in the senescent CNS [30, 31], and may further deteriorate the already poor regenerative outcome. Notably, also the immune system is subjected to aging [32–34], which may complicate its functioning during regeneration after CNS injuries. Despite its relevance, the impact of immune senescence on the regenerative capacity in the aged CNS remains poorly understood. Also in zebrafish, which has recently been established as a valuable model for human CNS aging [35], in-depth characterization of the interplay between acute inflammation and axonal regeneration in an aging context is still lacking.

This review aims at providing an overview of the current understanding of how inflammatory factors modify the regenerative outcome after damage in the adult CNS, in both mammals and zebrafish, thereby focusing on microglia and macrophages. Moreover, the effect of aging on inflammatory cell physiology and the implications this may have on the regenerative capacity will be discussed.

## 2. Acute Inflammation Promotes Axonal Regeneration in Mammals

Over the past decades, intensive research efforts have focused on the discovery of novel targets of which manipulation could enable regeneration after CNS trauma. Modulation of the acute inflammatory response has been proposed as promising strategy to induce axonal regeneration. Compelling evidence for a beneficial influence of different aspects of neuroinflammation has been gathered in various brain injury models [17, 36, 37]. Here, we will focus on the role of inflammation during axonal regeneration after optic nerve and spinal cord injuries.

### 2.1. Inflammatory Stimulation Improves the Regenerative Outcome

Stimulation of inflammation has proven to be one of the pivotal factors to induce a regenerative response in mammalian axonal regeneration models. For optic nerve injury, early studies have shown that a peripheral nerve graft, lens injury, or intravitreal injection of zymosan, a proinflammatory glucan molecule derived from the yeast cell wall, can induce RGC axon growth in rodents [38–41]. More recently, the smaller molecule Pam_3_Cys, which acts on the Toll-like receptor 2 (TLR2), is shown to stimulate axonal regeneration as well [42, 43]. All these experimental procedures induce activation of retinal micro- and macroglia and are accompanied by an influx of neutrophils and macrophages to the vitreous. Therefore, these treatments can collectively be referred to as “inflammatory stimulation” [4, 43].

Similarly, inflammatory stimulation improves the regenerative outcome after spinal cord injury. For example, intraspinal injection of zymosan increases axon growth [44], and stimulation with Pam2CSK4, another TLR2 agonist, was found to reduce axonal loss after spinal cord injury. This neuroprotection is a prerequisite for growth cone formation and subsequent axonal regeneration [45]. Thus, multiple lines of evidence point towards a positive effect of inflammation on (neuroprotection and) axonal regeneration of damaged neurons. However, in order to gain a better understanding of this process, detailed characterization of the nature of inflammatory mediators is indispensable.

### 2.2. Mediators of Inflammation in Optic Nerve Regeneration

#### 2.2.1. Inflammatory Events at the Injury Site

Similar to other CNS lesions, acute damage to the optic nerve causes changes in the microenvironment directly surrounding the site of the injury. Myelin, which is normally wrapped around the axons, becomes fragmented, leaving the axon tips exposed to myelin-derived inhibitory molecules such as Nogo, myelin-associated glycoprotein (MAG), and oligodendrocyte-myelin glycoprotein (OMgp) [4, 7]. Very soon after the injury, resident microglia become reactivated and monocyte-derived macrophages are recruited to the lesion site. Microglia are involved in the reactivation of astrocytes, as they start secreting various cytokines and other factors [46]. Eventually, these glial cells all contribute to the formation of scar tissue, which represents an important barrier to regenerating axons [4, 47]. Furthermore, the actions of microglia/macrophages as well as astrocytes have been suggested to lead to the propagation of secondary degeneration, which contributes to spreading of damage beyond the initial (primary) lesion site [48, 49]. Alternatively, microglia/macrophages have also been suggested to exert beneficial functions at the lesion site, such as phagocytosis of myelin debris or protection against glutamate excitotoxicity. The outcome of microglial/macrophage activation, whether positive or negative, is supposed to be highly dependent on the timing and the precise pathological conditions [49, 50] and thus needs further elucidation.

#### 2.2.2. Inflammatory Events in the Retina

Injury to the optic nerve induces acute inflammatory processes not only at the epicenter of the lesion, but also in the retina, where the RGC cell somata reside. Since stimulation of inflammation in the optic nerve as well as in the eye can protect RGCs, but only the latter promotes axonal regeneration [51], paradigms of inflammatory stimulation focus on altering retinal events.

After optic nerve injury, the resident retinal microglia respond rapidly, which can be considered a primary event resulting from the injury. However, microglial reactivation in the retina has also been linked to secondary degeneration of RGCs, although its exact role is unclear [49]. Yet, microglial reactivation does not occur uniformly across the retina. In the naive adult retina, surveying microglia are mainly found in four layers: the nerve fibre layer (NFL), the ganglion cell layer (GCL), and the inner and outer plexiform layers (IPL and OPL, resp.) [52, 53]. Upon injury, microglia in the OPL remain almost unaffected, while their cell number is increased and morphology is switched from ramified to amoeboid in the inner retinal layers, and most prominently in the GCL [53, 54]. In adult rats, microglial cell numbers augment dramatically from 3 days to 3 weeks after injury and return to almost normal levels by 6 weeks [53]. This increase coincides with the period during which RGC death occurs in response to the injury [5, 52, 53], and microglia actively phagocytize the debris from these RGCs and their axons [52–54]. Notably, the increase in glial cell number after optic nerve injury may either originate from local proliferation or from infiltration of blood-derived macrophages [53], but as it is very difficult to discriminate between these two cell populations [24], the relative contribution of both processes remains unclear.

Interestingly, microglial activation is also observed in the contralateral, uninjured eye. Although the increase in microglial density is not as high as in the ipsilateral retina and is mostly confined to the central retina, clear morphological changes can be observed [52, 53]. This suggests microglial activation in both eyes upon unilateral optic nerve damage, pointing towards cross-talk between both eyes, which may be orchestrated via the optic chiasm or hematogenous transference [53, 55]. Yet, its physiological function remains to be elucidated. Of note, this finding then also clearly indicates that the use of the contralateral eye as an internal control in retinal de- and regeneration studies should be reconsidered [53].

Next to the reactivated amoeboid microglia, another unique, so-called rod microglial morphology has recently been described in the mouse retina after optic nerve injury [56]. Rod microglia are first discovered after injury in the brain cortex [57] and are also described in the retina in a laser-induced ocular hypertension model for glaucoma [58, 59]. After optic nerve injury, the rod microglia are present from day 7 and are completely gone by 6 weeks. They are suggested to form the major group of phagocytic cells in the retina during this time [56].

**Figure 1:**
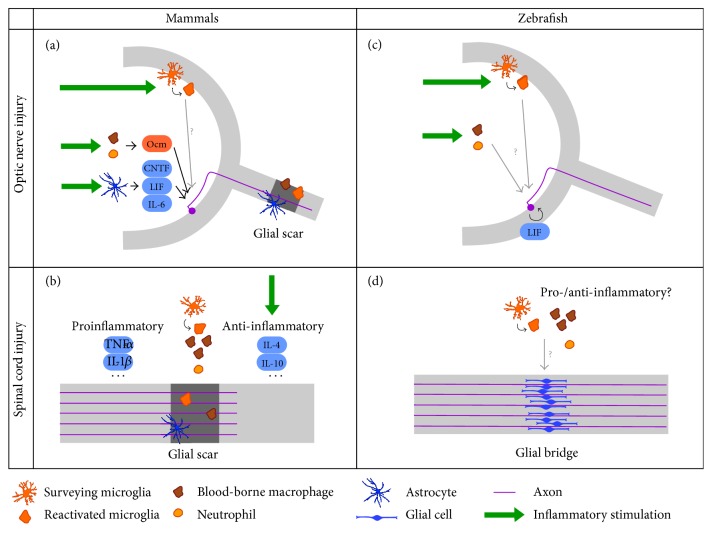
Summary of the current knowledge of the role of acute inflammation in axonal regeneration, in mammals and zebrafish. (a) After optic nerve injury in mammals, surveying retinal microglia become reactivated, proliferate, and transform into amoeboid microglia. Inflammatory stimulation (green arrows), which can be achieved via administration of TLR2 agonists or lens injury, further induces micro- and macroglial cell activation and an influx of neutrophils and blood-borne macrophages to the vitreous. Infiltrating macrophages and neutrophils secrete oncomodulin (Ocm), an inflammatory mediator that is thought to act on RGCs directly. Moreover, inflammatory stimulation elicits the secretion of IL-6 family cytokines from reactive astrocytes. Signal transduction of these cytokines is primarily mediated via the JAK/STAT3 and mTOR pathways in RGCs. Thus, inflammatory stimulation activates the intrinsic growth state of RGCs, and when combined with SOCS3 and/or PTEN deletion, feedback inhibitors of the JAK/STAT3 and mTOR pathway, respectively, axon regeneration beyond the glial scar can be obtained. (b) After an injury in the mammalian spinal cord microglia are activated, and neutrophils and blood-borne macrophages are recruited to the lesion site. Microglia/macrophages mostly adopt the proinflammatory phenotype and secrete proinflammatory cytokines such as TNF-*α* and IL-1*β*, while anti-inflammatory microglia/macrophages, which produce anti-inflammatory cytokines including IL-4 and IL-10, only represent a small percentage. Since the proinflammatory type is associated with adverse effects on regeneration, while anti-inflammatory cells are assumed to be protective and growth-promoting, treatments that stimulate anti-inflammatory activation at the expense of the proinflammatory type (green arrows) improve axonal growth beyond the glial scar and coincide with a better regenerative outcome. (c) Stimulation of acute inflammation after optic nerve injury in zebrafish activates microglia and induces recruitment of neutrophils and blood-borne macrophages, mirroring the situation in mammals. This results in an acceleration of the spontaneous regenerative process. Although it has already been shown that LIF and the JAK/STAT3 and mTOR pathways are implicated in optic nerve regeneration in zebrafish as well, the precise mechanism of how the positive effects of acute inflammation is mediated remains elusive. (d) After a spinal cord injury in zebrafish, microglia are activated and neutrophils and blood-borne macrophages infiltrate the lesion site, although their precise contribution to axonal regeneration is still unknown. Despite pro- and anti-inflammatory macrophages have been described in zebrafish injury models outside the CNS, the polarization of microglia/macrophages after spinal cord injury has not yet been studied. Strikingly, a growth-permissive glial bridge is formed at the lesion site, while glial scar is absent in zebrafish.

The functional role of resident microglia in neuroprotection and axonal regeneration after optic nerve injury has not yet been studied in detail. Instead, most attention has been given to the role of retinal macroglia (mostly astrocytes) on the one hand and infiltrating leukocytes (neutrophils and macrophages) on the other hand. Nevertheless, some debate exists on their relative importance. Firstly, axonal regeneration can be stimulated via intravitreal injection of zymosan, which causes the infiltration of neutrophils and macrophages. These cells may in turn serve as a source for oncomodulin, a small calcium-binding protein that has been suggested to be one of the major mediators of the beneficial effect of the intraocular inflammation on axonal regeneration (Figure 1(a)). Its function is dependent on the presence of cAMP and mannose [60–62] and thought to result from its binding to a high-affinity receptor on RGCs [60, 63]. Administration of a peptide competing for oncomodulin receptor binding was found to prohibit axon growth after optic nerve injury in mice [16, 61, 63]. Furthermore, combined deletion of* dectin-1* and* TLR2*, both coding for pattern recognition receptors expressed by inflammatory cells and necessary to respond to inflammatory stimulation, completely abolishes the regeneration-promoting effect of zymosan, again pointing towards the importance of immune mediators in axonal regeneration [64].

Second, inflammatory stimulation also induces the release of cytokines from activated retinal macroglia. Three cytokines from the IL-6 superfamily, namely, ciliary neurotrophic factor (CNTF), leukemia inhibitory factor (LIF), and IL-6, have been proposed as key mediators of the stimulating effect of inflammation on optic nerve regeneration [4, 65, 66] (Figure 1(a)). Indeed, the neuroprotective and axon growth stimulating effects of inflammatory stimulation are diminished in CNTF knock-out mice and abolished in CNTF/LIF double knock-out mice, thereby ascribing a principal role to CNTF, with LIF as an additional contributing factor [65]. Of note, direct intravitreal administration of CNTF has only limited effects on axonal regeneration, yet this might be explained by the short half-life of the protein in the vitreous [40, 67, 68]. The continuous release of CNTF and LIF by astrocytes after inflammatory stimulation, ensuring prolonged supply to RGCs, can be mimicked via AAV-mediated expression of CNTF in RGCs [69–71] or in Müller glia [72]. This viral CNTF expression has stronger effects on neuroprotection and axonal regeneration than those achieved via intravitreal injection, eliciting long-distance regrowth of axons up to the optic chiasm, but rarely beyond [1, 72]. Altogether, these studies clearly indicate that this group of cytokines plays a determining role in optic nerve regeneration. To date, however, the relative importance of oncomodulin, CNTF, LIF, IL-6, and other inflammatory factors has not yet been fully elucidated.

The importance of acute inflammation as a positive determinant in optic nerve regeneration is also reflected in some of the downstream molecular mechanisms and pathways identified thus far. Signal transduction of IL-6 superfamily cytokines is primarily mediated via the Janus Kinase/Signal Transducers and Activators of Transcription (JAK/STAT) pathway, which has been identified as an important positive player in optic nerve regeneration. The protein suppressor of cytokine signaling 3 (SOCS3), a feedback inhibitor of the JAK/STAT pathway, has counteracting effects on axon regeneration after optic nerve injury. Accordingly, deletion of the SOCS3 gene markedly enhances axon growth and improves the regenerative outcome of intravitreal CNTF administration [4, 10, 73]. Consistently, AAV-mediated overexpression of SOCS3 in RGCs almost completely abolishes RGC regeneration and suppresses the otherwise neurotrophic effect of intravitreal CNTF administration [74]. Of note, it has been shown that SOCS3 expression can be counteracted be delivering cAMP [75], which might explain the positive effect of elevating cAMP levels on axonal regeneration induced by inflammatory stimulation [4, 76]. Deletion of SOCS3, combined with a deletion of phosphatase and tensin homolog (PTEN), an upstream inhibitor of the mammalian target of rapamycin (mTOR) pathway that is also repeatedly shown to inhibit axonal regeneration, whether or not in combination with inflammatory stimulation [76–78], has been reported to induce a remarkable regenerative response [8–10, 76]. One study that uses PTEN deletion in combination with zymosan and a cAMP analog even reports scanty reinnervation of visual brain areas, including the lateral geniculate nucleus and the superior colliculus, and partial visual recovery [8]. Recently, it has also been shown that enhancing neural activity of RGCs via visual stimulation or chemogenetics, in combination with stimulation of mTOR activity by overexpressing the positive regulator Ras homolog enriched in brain 1 (Rheb1), enables long-distance and target-specific RGC axonal regeneration. This is accompanied by partial restoration of visual function [79]. Furthermore, continuous AAV-driven expression of hyper IL-6 cytokine (hIL-6), a designer cytokine that consist of the covalently linked bioactive parts of IL-6 and IL-6R*α*, in RGCs has been recently described as the most potent unifactorial treatment to promote axonal regeneration known thus far. When combined with PTEN deletion, hIL-6 improves optic nerve regeneration even more, with some axons reaching the chiasm within 3 weeks after optic nerve injury [78]. Conclusively, novel therapeutic approaches based on recent insights in the beneficial role of inflammatory mediators in regenerative processes hold exciting promise.

### 2.3. Mediators of Inflammation in Spinal Cord Regeneration

#### 2.3.1. Inflammatory Events at the Injury Site

Similar to other CNS injuries, damage to the spinal cord is followed by an acute inflammatory response. Resident microglia are activated, and neutrophils, macrophages, and lymphocytes infiltrate the lesion site. Also here, this inflammatory reaction eventually becomes chronic, and reactive astrocytes form a regeneration-inhibiting glial scar [19, 80, 81]. In the search for the exact contribution of inflammation to the regenerative potential, most studies in the field of spinal cord regeneration have focused on the role of microglia and blood-borne macrophages. Resident microglia are the first to respond to the injury, and infiltrating macrophages reach the injury site during the following days [82–86]. Most of these monocyte-derived macrophages originate from the spleen, while only a minority is mobilized from the bone marrow reservoir [86].

It has been proposed that different states of microglia/macrophage activation may influence the repair process [87]. Indeed, monocyte-derived macrophages polarize into different phenotypes, which are determined by the microenvironment and may change in response to new stimuli [88]. These functional states are generally divided into two main classes, based on the activation pathway, known as the “classically activated” proinflammatory M1 macrophages and the “alternatively activated” anti-inflammatory M2 macrophages. Later, additional subtypes of M2 (M2a, M2b, and M2c) have been described. However, macrophage activation is far more diverse than these simple categories. As such, the M1/M2 phenotypes rather represent two extreme poles, with in between a whole spectrum of activation states with overlapping properties [89–92]. A similar polarization has also been described for microglia [18]. Although there is now a general consensus that this M1/M2 classification of microglia/macrophage activation is an oversimplification, it nevertheless persists as a conceptual framework to facilitate our understanding of microglia/macrophage function [93].

After spinal cord injury, proinflammatory, M1-like macrophages are associated with secondary tissue damage, neuronal loss, axon retraction and demyelination, while anti-inflammatory, M2-like macrophages are assumed to be protective and promoting axon growth. In this regard, the balance between pro- and anti-inflammatory macrophages could be a major factor determining the regenerative outcome [19, 23]. Indeed, it has been demonstrated that most microglia/macrophages in the injured spinal cord display an M1-like activation state, with only a limited and transient presence of M2-like cells. Moreover, evidence suggests that lesion-derived factors (cytokines, chemokines, etc.) affect the microglial/macrophage phenotype, thereby favouring the proinflammatory state [94]. Therefore, it has been hypothesized that shifting macrophage activation towards the anti-inflammatory state may improve the regenerative outcome, mirroring successful tissue repair responses such as those occurring after skin or muscle injuries [87]. Some recent studies have indeed provided evidence for the beneficial effect of an augmented number of M2-like macrophages. For example, transfer of in vitro polarized M2-like macrophages to the damaged spinal cord improves functional recovery. Notably, this transfer of M2-like cells is suggested to alter the local microenvironment, thereby promoting the anti-inflammatory state [95]. Moreover, blocking of the IL-6 signaling pathway, via inhibition of the IL-6 receptor, results in an increase in M2-like microglia/macrophages at the expense of the M1-like type. Indeed, this treatment inhibits classical activation and promotes the alternative pathway and is accompanied by an improved functional recovery [96]. Taken together, these studies suggest that promoting alternative microglial/macrophage activation is a promising strategy to induce spinal cord regeneration (Figure 1(b)). Of note, it has been argued that activated microglia and monocyte-derived macrophages form functionally distinct, nonredundant cell populations after spinal cord injury and do not contribute equally to the repair process. As such, it has been suggested that microglia rather than infiltrating macrophages unequivocally express markers for the pro- or anti-inflammatory phenotype [86]. Moreover, the beneficial secretion of the anti-inflammatory cytokine IL-10 is attributed to (a subset of) infiltrating macrophages but cannot be provided by the activated resident microglia [24]. Yet, the relative contribution of microglia and macrophages still needs further clarification.

#### 2.3.2. Inflammatory Events at the Cell Soma of the Injured Neurons

Similar to the visual system, one should not only consider acute inflammation at the injury site itself but also turn to the cell soma of the axotomised neurons. However, the spinal cord is a much more complex structure than the optic nerve. While the latter only consists of RGC axons, the spinal cord is a well organised structure comprising neurons and axons of different types [97]. The cell bodies of the axotomised axons after spinal cord injury thus reside at different locations, challenging an unequivocal study of the inflammatory events that are provoked there. It has been repeatedly shown that spinal cord injuries induce widespread microglial activation in different brain areas, also outside the regions where the cell bodies of descending axon tracts are located [98–100]. However, this has mostly been associated with cognitive impairments and neuropathic pain as a result of the injury and not yet to regenerative processes. Thus, whether modification of these inflammatory responses would contribute to enhanced regeneration of the spinal cord remains elusive.

## 3. Successful Coupling of Neuroinflammation and CNS Regeneration in Zebrafish

Since adult zebrafish are capable of functional recovery after many CNS injuries, including optic nerve and spinal cord damage [27], they provide an attractive approach to study the interplay of inflammatory processes and CNS repair in a regenerative-supporting setting. Research in mammals may benefit from comparative studies in zebrafish. Amongst other advantages, this species provides an example of how acute inflammation can be linked to successful axonal regeneration. Importantly, the zebrafish immune system is highly comparable to its mammalian counterpart. The major immune cell lineages have been identified in zebrafish, and many of the immune receptor classes, signaling pathways, and inflammatory mediators are conserved as well [101].

### 3.1. Acute Inflammation in Zebrafish Optic Nerve Regeneration

Also in zebrafish, optic nerve injury models are well established. However, in contrast to mammals, in which a vast number of RGCs undergo apoptosis after optic nerve injury [5, 102], the large majority of their zebrafish counterparts seem protected and do survive the lesion [103, 104]. In a subsequent regenerative process, the damaged RGCs regrow their axons and reinnervate the target areas in the brain, of which the optic tectum is by far the most important [105–107]. The reestablished synaptic contacts are remarkably accurate and visual function eventually recovers, as shown by means of various vision-guided behavioural assays [27, 104].

Although the flawless regenerative responses after optic nerve injury in zebrafish are well known, the underlying cellular and molecular bases are less well understood. Nevertheless, analysis of gene expression patterns after optic nerve injury has already provided a framework for further functional studies [108, 109], and recent efforts have started to uncover the regulating mechanisms (recently reviewed by [110]). Noteworthy, no glial scar tissue is formed at the injury site. This may be attributed to the presumed absence of astrocytes in zebrafish. Instead, radial glial cells are assumed to take over at least part of the functions of mammalian astrocytes throughout the zebrafish CNS. Thus, the absence of a regeneration-inhibiting glial upon injury, may to some extent be explained by the functional differences between mammalian astrocytes and zebrafish radial glia [111].

**Figure 2:**
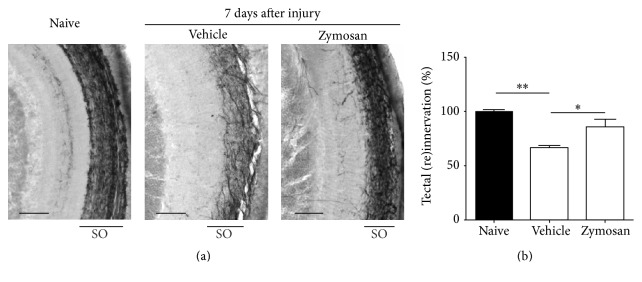
Intravitreal injection of zymosan accelerates axonal regeneration in zebrafish. (a) Representative images of biocytin-labeled axons in the contralateral optic tectum of naive fish (left) and fish treated with vehicle or zymosan, at 7 days after optic nerve injury (middle and right, resp.). The stratum opticum (SO), the layer through which the RGC axons innervate the tectum, is indicated. Scale bar = 50 *µ*m. (b) Quantification of the reinnervated area of the optic tectum in fish treated with vehicle or zymosan at 7 days after injury, relative to naive fish. Intravitreal injection of zymosan significantly accelerates reinnervation, which is already close to naive levels in zymosan treated fish as compared to vehicle-injected fish. Data represent mean ± SEM (*n* ≥ 3 animals per group, ^*∗*^*p* < 0.05, and ^*∗∗*^*p* < 0.01).

Of importance, there is evidence to support the hypothesis that acute retinal neuroinflammation might provide the right context for the initiation of a regenerative response. Firstly, an acute inflammatory response is observed after optic nerve crush in fish, which has been suggested to positively contribute to regeneration. More specifically, the number of microglia/macrophages in the retina increases significantly from 3 days after optic nerve crush onwards but is resolved around 3 weeks after the injury [103]. Furthermore, intravitreal application of zymosan, which results in a massive number of neutrophils and microglia/macrophages in the retina, efficiently stimulates optic nerve regeneration in zebrafish [103] (Figure 1(c)). Of note, application of zymosan has been associated with folding and retinal detachment of the mouse retina [112]. Although this cannot completely be ruled out, these effects have not been reported in zebrafish. In our laboratory, we have been able to confirm the positive effect of intravitreal zymosan injection on the regenerative process. Zymosan administration 3 days prior to optic nerve injury highly stimulated RGC axonal regeneration, assessed at 7 days after injury via anterograde biocytin tracing as described previously [113, 114]. Indeed, reinnervation of the optic tectum was found to be significantly increased in the zymosan-treated group, indicating that inflammatory stimulation accelerated the regenerative response after optic nerve injury in zebrafish (Figure 2). Thus, also in zebrafish inflammatory cells seem to have extensive effects on regeneration.

Second, recent evidence indicates that also in zebrafish the IL-6 cytokine superfamily stimulates axonal regeneration in an autocrine/paracrine manner, where especially LIF, rather than CNTF or IL-6, seems to be involved. LIF is upregulated upon injury in the fish retina and suggested to play a beneficial role in the early phase of the regenerative process [115, 116]. Strikingly and similar to mammals, endogenous expression of zebrafish Socs3a also counteracts regeneration after optic nerve injury, as is evidenced by enhanced RGC axonal regeneration after Socs3a knockdown. However, despite the activation of this inhibitory pathway, zebrafish still possess the ability of robust axonal regeneration [115]. Furthermore, inhibiting mTOR activity compromises optic nerve regeneration. This indicates a supportive role for mTOR, although it seems ancillary rather than essential for zebrafish axonal regeneration [117]. Clearly, factors counteracting regeneration are not absent in zebrafish, yet evolution seems to have provided them with a way to overcome these inhibitory mechanisms. The key to successful regeneration in mammals may thus reside in finding a proper balance between growth inhibition and stimulation [1, 110].

### 3.2. Acute Inflammation in Zebrafish Spinal Cord Regeneration

Models to study spinal cord regeneration are well developed in adult zebrafish, where, again, damage to the spinal cord is followed by a spontaneous regeneration process. After a complete spinal cord transection, a growth-permissive glial cell bridge is formed between the rostral and caudal lesion site [118] and, by two weeks after injury, cerebrospinal axons have started to regenerate beyond the transection site. This axonal regeneration correlates with functional recovery, and most fish regain their swimming abilities by five to eight weeks after injury [27, 119]. Of note, the regenerative potential is not equal for all axons in the spinal cord, as some axon types show only poor regrowth [27]. Apart from axonal regeneration, regenerative neurogenesis has also been described after spinal cord injury in zebrafish. Motor neurons and different types of interneurons are generated from radial glial cells in the region adjacent to the injury site [120]. It has been demonstrated that some newly generated motor neurons may even be capable of connecting with their peripheral muscle targets, indicative of effective integration into the existing spinal network [121]. However, since this only applies to a small number of newborn motor neurons, it remains uncertain whether regenerative neurogenesis significantly contributes to functional recovery [122].

Damage to the zebrafish spinal cord induces an acute inflammatory response, including the activation of microglia and monocyte-derived macrophages [123, 124]. However, its functional contribution to the regenerative process remains largely unexplored [125] (Figure 1(d)). One study reports that lysophosphatidic acid has proinflammatory but antiregenerative effects after spinal cord injury, in zebrafish as well as in mice [126]. However, this does not completely rule out the possibility of a beneficial role of inflammatory cells, since their phenotype can differ upon different types of stimulation, as described above. Besides, it has been demonstrated that zebrafish Ptena negatively affects regeneration after spinal cord injury, a finding that mirrors the observations in mammals [127] and can be linked to inflammatory pathways, as described above. Lastly, in a model of motor neuron ablation in larval zebrafish, in which motor neurons regenerate from spinal progenitor cells, the microglia/macrophages that gather at the lesion site are suggested to play a beneficial role during regeneration. Indeed, suppressing the immune response via treatment with dexamethasone, a synthetic glucocorticoid with anti-inflammatory effects [128] significantly suppresses motor neuron regeneration [129]. Yet, in-depth characterization of the role of acute inflammation in zebrafish spinal cord regeneration is lacking.

### 3.3. Acute Inflammation in Other Zebrafish Regenerative Models

Neuroinflammation has been put forward as an important underpinning of successful regeneration in other zebrafish CNS injury models as well. In contrast to mammals, in which a stab injury in the CNS is followed by massive neuronal cell death, reactive gliosis, and the eventual formation of a growth-inhibiting glial scar [28, 130–132], zebrafish can recover from such a lesion. Although cell death and reactive proliferation of microglia, oligodendrocytes, and other cells are observed in zebrafish as well, this resolves quickly and no evidence of glial scar tissue can be found [28, 29, 132]. Furthermore, zebrafish are able to initiate massive regenerative neurogenesis to compensate for the lost neurons. These newly generated neurons derive from proliferating radial glia cells [133]. Importantly, it has been proposed that acute neuroinflammation acts as a beneficial regulator of de novo neurogenesis in zebrafish. Systemic treatment with dexamethasone reduces the number of microglia/macrophages at the lesion site, coinciding with a diminished proliferation of radial glia and less newborn neurons, thus preventing regeneration after stab injury to the telencephalon [29]. Furthermore, the leukotriene signaling pathway appears to be a pivotal component of the inflammatory reaction and leukotriene C4 (LTC4), which signals through the cysteinyl leukotriene receptor 1 (CysLT1), is found to be necessary and sufficient for the initiation of neural progenitor proliferation and subsequent neurogenesis. Conclusively, inflammation appears to positively affect the reparative capacities in the zebrafish CNS [28, 29, 134, 135].

### 3.4. Macrophage Polarization in Zebrafish

Recently, M1- and M2-like macrophage subsets have also been observed in zebrafish. They express some of the markers that are typical for their mammalian counterparts. After caudal fin amputation or bacterial infection in larval fish, these macrophage phenotypes are activated in a time-dependent manner. Mirroring successful wound healing in mammals, monocyte-derived macrophages are recruited to the site of injury, where they first adopt a proinflammatory M1-like phenotype. In a later stage, this phenotype is progressively converted to M2-like, encompassing intermediate phenotypes in which both M1 and M2 markers are expressed [136]. This sequential M1-M2 response is supposed to be optimal for regeneration and contrasts the overwhelming presence of proinflammatory M1 microglia/macrophages observed after CNS injuries in mammals. Although supporting evidence from other zebrafish regeneration models is still needed, this study provides a first indication of functional similarities of microglia/macrophages in zebrafish and mammals.

## 4. The Impact of Inflammaging

To date, most studies aiming at the induction of CNS regeneration in mammals have been performed in young adult animals. However, the effect of aging on the regenerative mechanisms should not be overlooked. The limited regenerative capacity of the adult mammalian CNS further declines upon aging, and aging processes compromise the implementation of therapeutic strategies [137]. This is a major concern, especially since aging is one of the most important risk factors for many neurodegenerative diseases, and regenerative therapies are therefore most needed in elderly. Importantly, the immune system is subjected to aging as well, adding another level of complexity to this issue. Here, we will focus on the physiology of microglia and blood-borne macrophages in an aging context, and the consequences for repair after CNS injury.

### 4.1. Inflammaging in Mammals

It is well recognized that mammalian aging is accompanied by a low-grade, chronic proinflammatory state, which is also referred to as “inflammaging” and can be considered as a manifestation of immunosenescence [34, 138, 139]. Inflammaging is a systemic phenomenon that also affects the CNS. In the rodent retina, it is associated with morphological changes and functional impairments of microglia [32, 140]. This includes a slight but significant increase in microglial cell density, which is also observed in some other brain regions [141, 142], but not in all [143, 144]. One explanation for this phenomenon might be a reduced functionality of individual microglia, resulting in the necessity for more cells [32, 145]. Secondly, the ordered distribution of microglia throughout the retina seems to be deteriorating upon aging: they migrate from the inner retina towards the periphery and accumulate in the subretinal space, which is normally devoid of microglia [141, 142]. In addition, aged retinal microglia show reduced arborisation and slower process motilities, which likely compromise their dynamic surveying behaviour, further suggesting functional defects [141]. As similar observations are made in other brain regions as well [146, 147], there is increasing evidence for an age-related decline in the ability of microglia to perform their normal tasks in the CNS.

In addition, microglia develop age-related changes in their immune physiology. Evidence suggests that they adopt an altered, more inflammatory status, associated with increased expression of activation markers and proinflammatory cytokines [32]. This phenomenon has also been referred to as microglial sensitization or “priming.” Indeed, the microglial responsiveness to stressors or damage cues from the local environment or the periphery is increased in aged individuals, and the resulting inflammatory reaction is more pronounced than in young adults [148, 149]. Thus, although seemingly contradictory, the functional impairments associated with microglial senescence may be accompanied by an exaggerated response to stress or injury because of microglial priming [149]. Of importance, these age-related changes in microglial function may render the CNS more vulnerable to neurodegeneration, but may also highly impact regenerative abilities [33, 150].

### 4.2. The Impact of Inflammaging on Mammalian CNS Regeneration

As a result of microglial priming, detrimental effects of the immune response upon injury may be promoted in aged individuals, thereby suppressing the beneficial aspects of inflammation and further restricting regenerative capacities. Some studies have already provided evidence for this hypothesis in different injury models. Firstly, after traumatic brain injury, the microglial response was indeed found to be more pronounced and prolonged in aged mice compared to young adults [151]. Another study shows that traumatic brain injury results in larger lesions in aged mice. Alongside, the immune activation is exaggerated, and an increased ratio of pro- to anti-inflammatory microglia/macrophages has been demonstrated [152]. Secondly, reduced functional recovery upon aging is also observed after spinal cord injury in mice and has been correlated with impairments in the induction of IL-4 receptor *α* (IL-4R*α*) expression in microglia. The authors suggest that the impaired recovery in aged mice is related to a reduced responsiveness of microglia/macrophages to IL-4 and thus a shift towards a proinflammatory cytokine activation [153]. Recently, this research group also demonstrated an age-related decline in macrophage IL-10 expression after spinal cord injury. Since IL-10 is a key indicator of M2-like activation, this points to a reduction in the number of anti-inflammatory macrophages, and thus a disrupted balance between macrophage phenotypes, which presumably underlies the observed impairment in functional recovery with age [154]. Lastly, age-related deteriorations of microglia have also been found after damage to the retina. In a laser-induced injury model, senescent microglia respond more slowly; that is, their process motility and migration rate are reduced. In addition, senescent microglia remain present at the injury site for a longer time period than in their young adult counterparts, indicating that resolution of the inflammatory reaction is retarded upon aging. Unfortunately, age-related differences in injury severity and functional recovery were not addressed in this study [141]. Conclusively, aging of microglia and blood-derived macrophages undoubtedly affects the regenerative capacity after CNS injuries.

### 4.3. The Effect of Inflammaging on the Zebrafish Regenerative Potential

Recently, the zebrafish has emerged as a valuable model for human aging. Substantial evidence points towards the presence of aging hallmarks in zebrafish, also in the CNS (reviewed by [35]). Therefore, it is an attractive model organism to shed light on the relationship between aging and regenerative capacities. An age-dependent decline in the regenerative capacity of the spinal cord has already been suggested [155], based on early findings in goldfish [156]. However, a comprehensive study of the effect of aging on axonal regeneration in the zebrafish CNS is still lacking, and the impact of inflammation herein has not yet been investigated. Currently, the only evidence that age-related alterations in the immune system may potentially underlie a diminished regenerative capacity in senescent fish comes from a model of optic nerve remyelination. Of note, in both rodents and zebrafish, the ability to restore myelin sheaths is high in young individuals but decreases upon aging. It has been suggested that after optic nerve demyelination in aged zebrafish, the reduced remyelination is a result of an impaired response of microglia/macrophages. Indeed, while microglia/macrophages are recruited to the lesion site in young adults at four days after injury, their number at this time point is not significantly different from the naive condition in aged fish [157]. Overall, although an association between altered inflammation and attenuated regeneration upon aging has already been put forward, the impact of (inflamm)aging on the normally flawless regenerative process in the zebrafish CNS is scarcely studied and awaits further in-depth characterization.

## 5. Conclusion

Over the past years, neurodegenerative diseases and CNS trauma have been a major focus on neuroscience research, with many studies dedicated to the elucidation of the cellular and molecular changes that underlie their pathology. The innate immune system is undoubtedly involved in the pathogenesis of many of these CNS conditions, yet increasing evidence suggests that it can also beneficially contribute to the regenerative process. Indeed, a balanced activity of inflammatory cell types, of which microglia and blood-borne macrophages are the most studied, has been shown to improve morphological and functional recovery after injury in optic nerve and spinal cord injury models.

Unlike mammals, zebrafish possess a powerful regenerative capacity after CNS lesions, which leads to successful repair and seems to coincide with a favourable inflammatory state. Further uncovering of the mechanisms that control inflammatory and regenerative processes might provide fruitful insights that may lead to the identification of innovative therapeutic targets for human patients.

Noteworthy, we foresee that an important hurdle will have to be taken in the development of novel CNS regenerative strategies from bench to bedside, being the fact that aging processes affect the already limited regenerative potential in mammals. Since the innate immune system is subjected to aging as well, it is assumed to react differently to injuries in aged individuals. Increasing evidence for a detrimental effect of inflammaging on the regenerative outcome is emerging, but further in-depth characterization in both mammals and zebrafish is highly warranted.

## Abbreviations

AAV: Adeno-associated virus
cAMP: Cyclic adenosine monophosphate
CNS: Central nervous system
CNTF: Ciliary neurotrophic factor
CysLT1: Cysteinyl leukotriene receptor 1
GCL: Ganglion cell layer
hIL-6: Hyper IL-6 cytokine
IL-4R*α*: IL-4 receptor *α*
IPL: Inner plexiform layer
JAK/STAT: Janus Kinase/Signal Transducers and Activators of Transcription
LIF: Leukemia inhibitory factor
LTC4: Leukotriene C4
mTOR: Mammalian target of rapamycin
NFL: Nerve fiber layer
OPL: Outer plexiform layer
PTEN: Phosphatase and tensin homolog
RGC: Retinal ganglion cell
Rheb1: Ras homolog enriched in brain 1
SOCS3: Suppressor of cytokine signaling 3
TLR2: Toll-like receptor 2
MAG: Myelinassociated glycoprotein
OMgp: Oligodendrocyte-myelin glycoprotein.
